# Certain Residuals of Epidemic of Cerebrospinal Meningitis Observed in the Army

**Published:** 1919

**Authors:** 


***Certain Residuals of Epidemic Cerebro-Spinal Meningitis Observed in the Army.*** By A. J. ROSANOFF, Major M. C., U. S. Army. *The Journal of the American Medical Association*, November 2, 1918.

The author records a series of postmeningitic cases observed at Camp Beauregard, La.

The symptoms were as follows :

1. Limitation of flexion of the spinal column.
2. Undue fatigability.
3. Pains in back, legs, and head.
4. Tendency towards dizziness and faintness.
3. Muscular weakness.
6. Tendency towards blurring of vision associated with photophobia.
7. Impairment of appetite and sleep associated with a state of under-nutrition.

As to headache, dizziness, faintness and loss of consciousness, the postmeningitic condition closely resembles the well-known condition that persists for years following severe cranial traumatism.

Muscular weakness is shown particularly, in the majority of cases, by feeble hand grips.

The tendency towards blurring of vision seems due to a weakness of the ocular muscles. Diplopia occurs in some cases, but, as a rule, the ocular movements are not impaired. In those cases in which the tendency to blurring of vision is most marked, there is also a degree of photophobia. In these cases there is sluggishness and limited excursion in the pupillary reaction to light.

The loss of sleep is almost invariably associated with pain.

The cases recorded have shown very considerable variation in severity of the symptoms and degree of disablement, which might be explained by the varying severity of infection and the patient's resistance to it. The patients were therefore divided into groups and placed under a regime of graded exercises, such as neck bending and body bending. Improvement followed within a month; in one case the patient was able to take part in a game of baseball.

The author speaks of the comparatively frequent occurrence of postmeningitic cases and of the little attention given to them. They have been treated as cases of hysteria, neurasthenia, psychoneurosis, etc., and men have been sent back to duty and even overseas, who were incompletely restored. The author therefore proposes that all patients with epidemic cerebrospinal meningitis should, on their recovery, be transferred to a designated army hospital for convalescence and such further observation and treatment as they may require.

